# A survey of population-based utility scores for cervical cancer prevention

**DOI:** 10.1186/1756-0500-7-899

**Published:** 2014-12-11

**Authors:** Leonardo Simonella, Kirsten Howard, Karen Canfell

**Affiliations:** Saw Swee Hock School of Public Health, National University of Singapore, 16 Medical Drive, Block MD3, Singapore, 117597 Singapore; School of Public Heath, University of Sydney, Edward Ford Building (A27), Sydney, NSW 2006 Australia; Cancer Research Division, Cancer Council NSW, Woolloomooloo, Australia; Lowy Cancer Research Centre, Prince of Wales Clinical School, The University of New South Wales, Kensington, Sydney, NSW 2052 Australia

**Keywords:** Economic evaluation, Health state utilities, HPV vaccination, HPV screening, Cervical screening

## Abstract

**Background:**

With human papillomavirus (HPV) vaccination introduced in a number of countries, there is considerable interest in evaluating the cost-effectiveness of HPV testing as the primary cervical screening test in these settings. However, the availability of utility scores for these newer interventions is limited. Our aim in this paper is to present utility scores for HPV vaccination, HPV testing and cytology based screening states among women targeted for cervical screening.

**Methods:**

We invited a random sample of women targeted for cervical screening (aged 20-69 years) living in Sydney, Australia, to participate in a face-to-face interview. Participants were asked to indicate preferences (rank and utility scores) for 10 hypothetical health states relating to HPV vaccination, cytology and primary HPV screening, cervical precursor disease and early stage cervical cancer. Preferences for hypothetical health states were measured through ranking then a two-stage standard gamble. Each participant’s own health state was measured as a utility score using the EQ5D. Potential differences by age were assessed using the Wilcox Rank Sum test.

**Results:**

A maximum of 276 women were contacted, of which 43 (mean age 49 years) agreed to be interviewed (15.6%). The overall health state of women as measured by the EQ5D was 0.86 (95% CI: 0.83-0.89). Of the 10 health states, the highest ranked were ‘normal cytology’ and ‘HPV vaccination’ (equal 1^st^). States involving an HPV positive result with a subsequent normal cytology or colposcopy were ranked below those for low grade cytological abnormalities with or without a subsequent colposcopic normal result (ranks 3-4 vs. 4-5). However, mean utility scores were broadly similar for all health states, except cervical cancer. No significant differences in scores were identified between age groups.

**Conclusion:**

Our survey suggests health states relating to HPV testing are ranked below ‘low grade cytology’ disease abnormalities. However, this difference was minimal on the utility scale, as most values for health states were largely clustered. These results provide a preliminary set of non-clinic population-based utilities that may be used with other values to explore the economic implications of introducing HPV testing as a primary screening tool in the context of HPV vaccination.

**Electronic supplementary material:**

The online version of this article (doi:10.1186/1756-0500-7-899) contains supplementary material, which is available to authorized users.

## Background

Over the last six years, a number of developed countries have introduced prophylactic vaccination against the sexually transmitted human papillomavirus (HPV) in pre-adolescent females. The decision to introduce HPV vaccination has been supported by results from randomised controlled trials and cost-effectiveness analyses (conducted in the context of existing cytology based cervical screening programs) [1]. At the same time, primary HPV DNA testing has emerged as an alternative screening technology to cervical cytology (‘Pap smears’), such that several randomised controlled trials in Europe and North America highlight higher sensitivity of primary HPV testing (i.e. testing for a bank of oncogenic HPV types) for detecting high grade precursor lesions (Cervical Intraepithelial Neoplasia grades 2 and 3 and above; CIN2+ and CIN3+) relative to cytology in baseline rounds of screening, with lower rates of these high grade precursor cervical lesions detected in subsequent screening rounds [2–6], as well as lower rates of invasive cervical cancer in HPV-screened women compared to cytology-screened women [5].

Due both to the emergence of these data on primary HPV testing and to the implementation of HPV vaccination, a number of countries are formally evaluating a transition to primary HPV screening. Consequently, a number of aspects of primary HPV testing will require detailed evaluation in cost-effectiveness models; these include the age range of women screened, the screening interval (in vaccinated and unvaccinated women), the triaging and management strategy for HPV-positive women, the role of partial genotyping systems, and the role of adjunctive co-testing (i.e. performing both HPV and cytology together at the primary screening stage).

Historically, decision analytic models used to evaluate the incremental cost-effectiveness of HPV vaccination in the context of existing cervical cytology screening programs have adopted a specific set of health state preference scores (utilities) to quantify the quality of life effects associated with cervical screening and any detected disease [7, 8]. However, a limitation of these scores is that they do not reflect the full complexity of health states that women may experience, nor do they provide preferences for interventions related specifically to primary HPV screening or HPV vaccination. Moreover, the study underpinning these scores did not assess preference across a representative age-sample of women targeted for cervical screening. This is important because the trade-off between benefits and harms of cervical screening potentially differs substantially between younger and older (>45-50 years) women, since there are some data to indicate that treatment for cervical precancerous abnormalities increases the risk of subsequent adverse obstetric outcomes in fertile women [9].

Our aim in this study was to estimate utility scores for health states related to HPV vaccination, primary HPV screening, cervical precursor disease and its treatment, and early stage cervical cancer, among women aged 18-69 years, which is the age group currently screened in Australia.

## Methods

### Study population

Participants were invited to participate via a regular population health survey conducted by the New South Wales (NSW) Department of Health, Australia [10, 11]. The state based health survey invites residents to take part in an over-the phone telephone interview. Participants are selected using a combination of random digit dialling to select eligible inhabitants within specific health service areas, followed by a letter of notification [11, 12]. Approximately 12,000 survey participants take part in the health survey, representing a response rate of 63.4% [10]. Women invited to participate in the current study were living in metropolitan Sydney and were within the specified age groups (20-49 years and 50-69 years). If the survey participant gave verbal consent, the NSW Department of Health passed on their contact details to the study investigators. Women aged 20-49 years were oversampled to enable appropriate representation in the survey. Women in younger age groups are generally oversampled due to their lower participation rate.

### Health state scenarios

Ten hypothetical cervical cancer prevention health states scenarios were evaluated in a face-to-face interview (Table 1). Each scenario was described in a narrative format to explain the process of investigation and treatment (if applicable), along with any physical and emotional consequences (see Additional file 1) [13]. The scenario descriptions were informed by relevant Australian screening and treatment guidelines as well as the psychosocial literature [14–17]. As primary HPV testing is not yet commonly used as part of routine screening in Australia, a scenario was constructed based on a review of screening options and quality of life assessments of experiences related to HPV testing [18, 19].

**Table 1:** Summary of hypothetical scenarios used to evaluate cervical cancer prevention health states*

Health state	Description ( ***character name in health state description*** )
Cytology normal	Pap test cytology negative (*Kelly*)
HPV vaccination	Three doses of the HPV vaccine (*Emily*)
LG cytology	Cytology screening with a low grade abnormality and a follow-up smear in 12 months (*Lisa*)
LG cytology with colposcopy normal	Cytology screening with a low grade abnormality and immediate colposcopy (*Andrea*)
HPV positive with cytology normal	HPV positive and cytology negative (to account for strategies involving either primary HPV screening with negative cytology triage or primary co-testing with HPV and cytology) (*Danielle*)
HPV positive with colposcopy normal	HPV oncogenic positive with an immediate colposcopy which has a normal result (to account for primary HPV screening involving partial genotyping for HPV 16, 18 and immediate referral of this group) (*Libby*)
Treated genital warts	Treatment for genital warts associated with HPV types 6 and 11 (*Angela*)
HG cytology with CIN 1	High grade abnormal cytology screening result with subsequent histologically-confirmed CIN Grade 1 (*Natalie*)
HG cytology with CIN 2 or 3	High grade abnormal cytology screening result with subsequent histologically-confirmed CIN Grade 2 or 3 (*Deborah*)
Early stage cervical cancer	Early stage cervical cancer requiring a hysterectomy (*Mary*)

* HPV human papillomavirus; LG low grade disease; HG high grade disease; CIN cervical intraepithelial neoplasia; FIGO Fédération Internationale de Gynécologie Obstétrique (International Federation of Gynecology and Obstetrics).

### Overview of interview process

Interviews were undertaken by a single interviewer (LS). After being given a brief description of the interview process to enable informed consent, the participant was provided with an explanation of concepts related to cervical cancer, HPV, the HPV vaccine and other cervical cancer prevention related activities, which was facilitated with visual displays and incorporated an opportunity for participants to ask questions. Participants then ranked the description of each health state by selecting, at random, one of the health states from the description vignettes; a second health state was then selected at random and ranked relative to the first; this process was continued until all ten health states were ranked relative to each other (equal ranking was permitted). The utility score for each of the 10 hypothetical health states was assessed using a modified version of the standard gamble (see below). The interview concluded by asking participants to document their age and own quality of life using the EQ5D™ instrument. Questions regarding whether a participant had experienced any of the health states was not requested at any time during selection for participation or during the interview process.

### Health state preference score assessment

**Figure 1:**
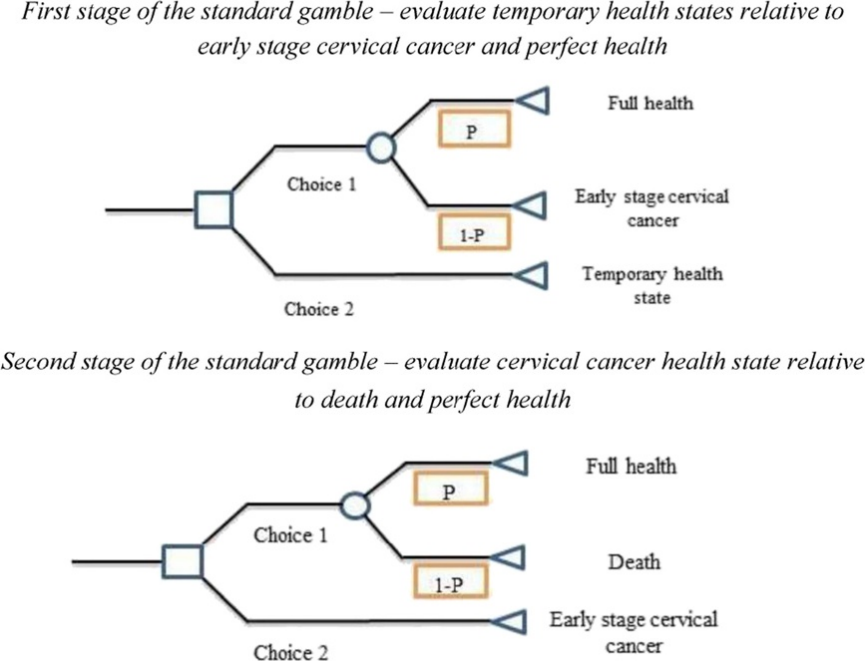
**Conceptual outline of two-stage standard gamble.**

A two-stage standard gamble was used to assess the utility for each hypothetical health state. The assessment process used two stages; Stage 1 derives probability indifference scores (utilities) for each of the nine health states, measured relative to risky prospects between perfect health and early stage cervical cancer, while Stage 2 derives a probability indifference score for early stage cervical cancer measured relative to the risky prospects associated with either perfect health or death (Figure 1) [20]. For stage 2, we evaluated cervical cancer twice using two distinct ‘time in state’ scales (see below). Thus each participant provided 11 health state preference scores; nine for each temporary health state, two for early stage cervical cancer. By valuing temporary health states that do not involve any likely prospect of death (i.e. the non-cancer states), ‘ceiling effects’ for the hypothetical health states are potentially minimised [21]. The two-stage standard gamble has been adopted in previous assessments of cervical cancer prevention [15, 22, 23].

For the temporary health states (non-cancer), participants were asked to imagine health returning to normal after 12 months (Stage 1). For the cervical cancer health state, where the probability of indifference between living with early stage cervical cancer is measured relative to the risky prospects associated with either perfect health or immediate death (stage 2), two ‘time in state’ durations for cervical cancer were used. The first was for 12 months followed by sudden and painless death, the second ‘time in state’ was to occur from the present until age 85 years followed by sudden and painless death. A 12 month period was used to correspond with the duration of the temporary health states, as is required for the two-stage standard gamble [20]. However, given women with early stage cervical cancer are unlikely to die within 12 months, a more clinical relevant duration was also used; that is, maximum life expectancy. The use of two ‘time in state’ durations for cervical cancer resulted in two sets of utility scores for each hypothetical health state. Differences in health states preference scores for each ‘time in state’ anchor point would provide an indication of whether hedonic load (period one has to live with the health state of interest) affects utility scores.

To determine the utility score for temporary health states on the 0 – 1 cardinal interval scale, the scores were mathematically transformed using the following function: *h*_*i*_ = *P*_*i*_ + (1 - *P*_*i*_)*h*_*k*_ where *h*_*i*_ is the utility of the temporary health state, *P*_*i*_ is the probability of indifference observed between the certain outcome of experiencing the temporary health state and the risky prospect of either living with early stage cervical cancer or living with perfect health, and *h*_*k*_ is the utility of early stage cervical cancer (worst health outcome) evaluated on the death to perfect health scale [20]. Thus, for early stage cervical cancer there are two *h*_*k*_; one evaluated on the 12 month time scale, and the other evaluated on the life time scale. For each individual utility score representing a temporary health state we applied two separate ‘time in state’ values representing the anchor state (using the mathematical function *h*_*i*_ = *P*_*i*_ + (1 - *P*_*i*_)*h*_*k*_. This resulted in two distinct utility scores on the 0-1 cardinal interval scale for each participant’s temporary health state, characterised by the ‘time in state’ value; yielding 18 temporary health state utility scores for each participant.

### Statistical analysis

Demographic characteristics (age and general health; as indicated by the EQ5D) were reported using mean and 95% confidence intervals (95% CI). Each utility score was summarised using the mean and standard error (SE) as well as the median and inter-quartile range (IQR). An intraclass correlation coefficient (ICC) was used to assess the level of agreement between each participants’ pair of utility scores transformed with the 12 month duration for ‘early stage cervical cancer’ and the lifetime duration [24]. The ICC was also used to measure the level of agreement between the pair of ‘early stage cervical cancer’ scores that were evaluated using the ‘12 month’ and ‘lifetime’ durations (‘time in state’ values). The ICC is calculated as (*MS*_*btw grps*_ - *MS*_*wthin grps*_)/(*MS*_*btw grps*_ + *MS*_*wthin grps*_); where *MS* refers to mean square. In our analysis the *MS*_*btw grps*_ is characterised as the difference between the grand mean for a health state (combining both sets of utility scores calculated using different ‘time in state’ values) and the group means for a health state calculated according for a specific ‘time in state’ value. Whereas *MS*_*wthin grps*_ is characterised as the difference between the specific ‘time in state’ individual utility scores and the mean of these scores. A low *MS*_*wthin grps*_ (relative to *MS*_*btw grps*_) will provide an ICC that is reasonable to good, whereas a high value will result in an ICC that is poor. For this analysis, we assumed ICC values of 0.80–1.0 to have ‘good’ agreement. ICC values between 0.50-0.80 were considered ‘reasonable’, while those less than 0.50 were considered ‘poor’. To enable calculation of ICCs, one-way analysis of variance (ANOVA) was carried out for each health state, such that mean square values (‘between groups’ and ‘within groups’) were determined for utility scores based on each ‘time in state’ anchor state.

An exploratory analysis to assess differences in mean utility scores for each temporary health state according to age (20-49 years vs 50-69 years) was conducted using the Wilcoxon Rank Sum Test [25]. Scores transformed with ‘12 month’ and ‘lifetime’ durations were analysed separately for each set of age group comparisons. Given we are testing 10 distinct health states, with two sets of ‘time in state’ anchor states for the mathematical transformation of temporary health states, a total of 20 tests for statistical significance were made. Consequently, the probability of making at least one ‘Type 1’ error (incorrectly rejecting a null hypothesis) is estimated to be around 64% (calculated from 1 - (1 - *α*)^*m*^: where *α* is the cut-off for statistical significance; 0.05, and *m* is the number of hypotheses tested; in this case 20), a Bonferroni correction of p < 0.003(defined as  was adopted as the ‘Type I’ error cut-off threshold to evaluate any statistically-significant difference between the age group for each utility score. All analyses were conducted in EXCEL.

### Ethical approval

The study was approved by the Human Research Ethics Committees of the University of Sydney and Cancer Council NSW, Australia.

## Results

### Study population

**Figure 2:**
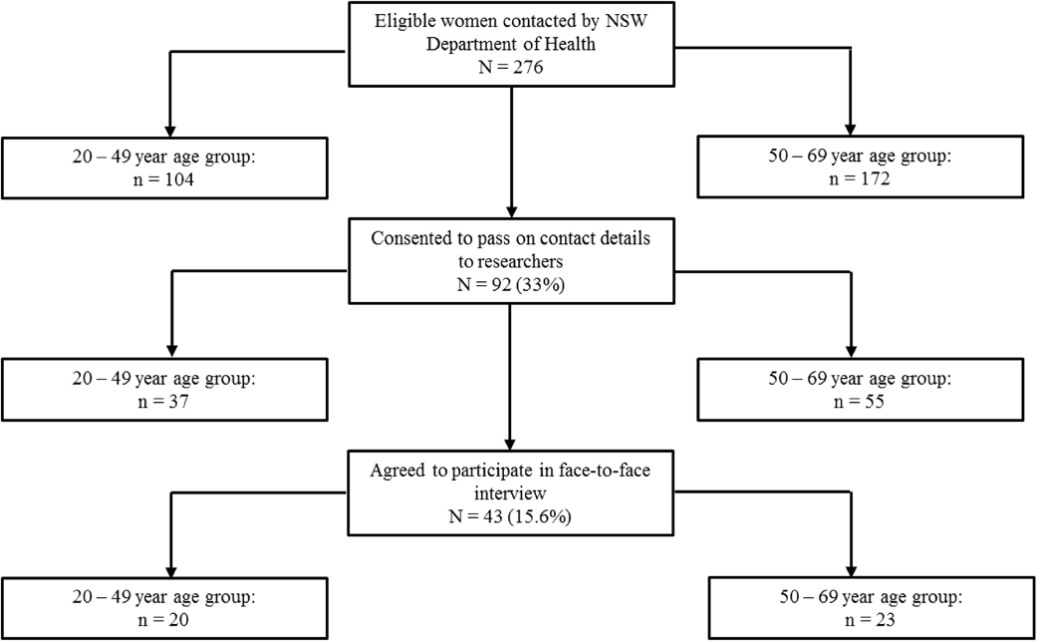
**Study participant flow chart - overall and by age group.**

Recruitment for the study took place between 25 October and 25 November 2010. A total of 276 eligible women were identified and contacted by the NSW Department of Health (Figure 2). Of these, 104 were aged 20-49 years, and 172 aged 50-69 years. Of the 276 eligible women, 92 (33%) gave consent to pass on their contact details to the investigators of which 37 were aged 20-49 years and 55 were aged 50-69 years. Of the 92 women who gave their consent to pass on their contact details, 43 (46.7%) agreed to participate.

Of the 43 participants, 20 were aged 20-49 and 23 were aged 50-69 years. The mean age of all participants was 49.4 years (95% CI: 45.6–53.2 years) (Table 2). Among the 20-49 year age group, the mean age was 38.2 years (95% CI: 34.6-41.8 years), while for the 50-69 year age group it was 59.1 years (95% CI: 56.7-61.3 years). The average overall EQ5D health score was 0.86 on the 0 to 1 scale of ‘worst imaginable health’ to ‘best imaginable health’ (95% CI: 0.83–0.89). The point estimate was slighter higher among the younger age group (0.89, 95% CI: 0.86–0.92) relative to the older age group (0.83, 95% CI: 0.79–0.87) (Table 2).

**Table 2:** **Health state evaluation measured using the EQ5D in the study population (mean and 95% confidence intervals)**

Characteristic	Overall	20-49 years	50-69 years
Age (years)	49.4	38.2	59.1
	*(45.6-53.2)*	*(34.6-41.8)*	*(56.7-61.3)*
Overall health*	0.86	0.89	0.83
(worst ‘0’ - best ‘1’ health)	*(0.83-0.89)*	*(0.86-0.92)*	*(0.79-0.87)*

### Overview of health state preference scores

Of the 43 participants interviewed, two (one from each age group) had difficulties with interpreting the standard gamble process. Therefore, results presented here are for the remaining 41 participants. The ordinal rank for each of the health states is presented in Table 3. The highest ranked health states were ‘Cytology normal’ and ‘HPV vaccination’ (median rank; 1; IQR; 1-2), while the lowest ranked was ‘early stage cervical cancer’ (median rank: 10; IQR: 8-10).

**Table 3:** **Rank, standard gamble utility scores and intraclass correlation coefficient for cervical cancer prevention health states**

		Cervical cancer duration				
Health state	Median rank	12 months		Lifetime		
		Mean utility score	Median utility score	Mean utility score	Median utility score	Intraclass correlation coefficient
	(IQR )	(SE)	(IQR)	(SE)	(IQR)	
Cytology normal	1	0.9967	0.9998	0.9995	1.0	0.13
	(1-2)	(0.0026)	(0.9994 - 1.0)	(0.0002)	(0.9998 -1.0)	
HPV vaccination	1	0.9750	0.9998	0.9978	1.0	0.07
	(1-2)	( 0.0244)	(0.9996 - 1.0)	(0.0018)	(0.9998 - 1.0)	
LG cytology	3	0.9735	0.9997	0.9980	1.0	0.05
	(3-5)	(0.0231)	(0.9994 - 1.0)	(0.0017)	(1.0 -1.0)	
LG cytology with colposcopy normal	4	0.9724	0.9997	0.9970	0.9999	0.08
	(3-6)	(0.0226)	(0.9964 - 0.9999)	(0.0017)	(0.9997 - 1.0)	
HPV positive with cytology normal	4	0.9733	0.9997	0.9970	0.9999	0.02
	(3-6)	(0.0233)	(0.9977 - 0.9999)	(0.0017)	(0.9997 - 1.0)	
HPV positive with colposcopy normal	5	0.9964	0.9997	0.9991	0.9999	0.22
	(3-6)	( 0.0021)	(0.9984 - 0.9999)	(0.0004)	(0.9997 - 1.0)	
Treated genital warts	5	0.9700	0.9997	0.9969	0.9998	0.09
	(3-7)	( 0.0244)	(0.9969 - 0.9999)	(0.0019)	(0.9997 - 1.0)	
HG cytology with CIN 1	6	0.9724	0.9997	0.9953	0.9999	0.01
	(4-8)	(0.0226)	(0.9970 - 0.9999)	(0.0027)	(0.9997 - 1.0)	
HG cytology with CIN 2 or 3	7	0.9704	0.9996	0.9970	0.9999	0.12
	(6-9)	(0.0233)	(0.9959 - 0.9999)	(0.0018)	(0.9994 - 1.0)	
Early stage cervical cancer	10	0.8178	0.9450	0.9714	0.9900	0.78
	(8-10)	(0.0531)	(0.915 - 0.995)	(0.0052)	(0.9450 - 0.9950)	

*HPV human papillomavirus; LG low grade disease; HG high grade disease; CIN cervical intraepithelial neoplasia; FIGO Fédération Internationale de Gynécologie Obstétrique (International Federation of Gynecology and Obstetrics.*

### Pap tests, HPV testing and HPV vaccination

Across the numerical descriptions of utility scores for each health state (‘12 month’ duration, mean; ‘12 month’ duration, median; ‘lifetime’ duration, mean; ‘lifetime’ duration, median), a ‘normal cytology result’ had a narrower range of utility scores compared with ‘HPV vaccination’ (0.9967 to 1.0 vs 0.9750 to 1.0), indicating greater heterogeneity in the evaluation of HPV vaccination state compared to the state relating to an experience of being screened with a normal cytology result (Table 3). For investigations involving an HPV test, a ‘positive for HPV infection with a normal cytology result’ had a lower range of preference scores relative to the health state described as ‘positive for HPV infection with a colposcopy normal result’ (0.9733 to 1.0 vs 0.9964 to 0.9999), indicating that more intensive investigations (colposcopy) resulted in higher utility scores.

### Low grade disease and genital warts

The health states described as ‘low grade cytology’ and ‘low grade cytology with a normal colposcopy result’ had similar ranges of scores (0.9735 to 1.0 vs 0.9724 to 0.9999) (Table 3). Although the state reflecting a high grade cytology result with subsequent confirmed CIN 1 involved an initial report of high grade cytology, on average, study participants assessed this to have the same utility score as a ‘low grade cytology with a normal colposcopy result’ (0.9724 to 0.9999 vs 0.9724 to 0.9999) (Table 3). The health state ‘treated genital warts’ had slightly lower range of health state preference score relative to the low grade disease health states (0.9700 to 0.9998).

### High grade disease and cervical cancer

The utility scores for ‘high grade cytology with confirmed CIN 2/3’ ranged from 0.9704 to 0.9999. The mean values for this health state (transformed using the ‘12 month-early stage cervical cancer’ score) were similar to the health state ‘treated genital warts’ (0.9704 vs 0.9700). Similarly, mean utility scores (transformed using the ‘lifetime duration-early stage cervical cancer’ score) were the same for a ‘low grade cytology with a normal colposcopy result’ and ‘positive for HPV infection with a normal cytology result’ (0.9970). The health state ‘early stage cervical cancer’ had the lowest utility score when assessed using 12 month ‘time in state’ (mean: 0.8178; SE: 0.05314, median: 0.9450; IQR: 0.9150-0.9950) compared with lifetime ‘time in state’ (mean: 0.9714; S.E: 0.005217, median: 0.9900; IQR: 0.9450-0.9950).

The intraclass correlation coefficient indicated reasonable agreement for ‘early stage cervical cancer’ utility scores assessed using a ‘12 month’ duration and a ‘lifetime’ duration (ICC: 0.78) (Table 3). By contrast, the level of agreement between non-cervical cancer health states converted on the 0-1 cardinal scale using the ‘12 month’ and ‘lifetime’ duration cervical cancer preference score was generally poor. These ranged from 0.01 (high grade cytology with confirmed CIN 1) to 0.22 (HPV positive with normal subsequent colposcopy). However, the absolute differences between the mean utility scores between each health state were small. For ‘high grade cytology with confirmed CIN 1: 0.9724 (using the 12 month duration cervical cancer value) minus 0.9953 (using the lifetime duration cervical cancer) equates to an absolute difference of 0.0229. For HPV positive with normal subsequent colposcopy the absolute difference was 0.0027.

Utility scores transformed using ‘12 month-early stage cervical cancer’ suggested no significant difference between women aged 20–49 and 50–69 years when assessed using the Wilcoxon Rank Sum Test (Tables 4 and 5). A similar pattern was observed for age group comparisons between utility scores transformed with ‘lifetime duration-early stage cervical cancer’ (Table 4).

**Table 4:** **Age-specific comparison of standard gamble utility scores converted using the ‘12 month-early stage cervical cancer’ score**

Health state	Age groups				Wilcoxan rank sum test P value
	20-49 years		50-69 years		
	Mean utility score	Median utility score (IQR)	Mean utility score	Median utility score	
	(SE)		(SE)	(IQR)	
Cytology normal	0.9938	0.9997	0.9990	0.9999	0.4932
	(0.0055)	(0.9994 - 1.0)	(0.0004)	(0.9994 - 1.0)	
HPV vaccination	0.9992	0.9998	0.9539	1.0	0.6534
	( 0.0004)	(0.9994 - 1.0)	( 0.0454)	(0.9996 - 1.0)	
LG cytology	0.9935	0.9997	0.9563	0.9998	0.2855
	(0.0055)	(0.9993 - 0.9999)	(0.0429)	(0.9996 - 1.0)	
LG cytology with colposcopy normal	0.9909	0.9997	0.9563	0.9997	0.3383
	(0.0070)	(0.9950 - 0.9999)	(0.0420)	(0.9969 - 1.0)	
HPV positive with cytology normal	0.9942	0.9997	0.9552	0.9997	0.3379
	(0.003955)	(0.9959 - 0.9999)	( 0.0434)	(0.9977 - 1.0)	
HPV positive with colposcopy normal	0.9934	0.9997	0.9990	0.9998	0.1280
	( 0.0045)	(0.9950 - 0.9999)	(0.0004)	(0.9989 - 0.9999)	
Treated genital warts	0.9894	0.9997	0.9532	0.9997	0.2483
	( 0.0056)	(0.9950 - 0.9999)	( 0.0454)	(0.9990 - 1.0)	
HG cytology with CIN 1	0.9956	0.9997	0.9523	0.9996	0.3725
	( 0.0024)	(0.9950 - 0.9997)	(0.0420)	(0.9977 - 0.9999)	
HG cytology with CIN 2 or 3	0.9908	0.9995	0.9527	0.9996	0.2826
	(0.0049)	(0.9948 - 0.9997)	(0.0433)	(0.9983 - 1.0)	
Early stage cervical cancer	0.7977	0.9450	0.8352	0.960	0.2616
	(0.0819)	(0.8850 - 0.9750)	(0.0709)	(0.915 - 0.995)	

*HPV human papillomavirus; LG low grade disease; HG high grade disease; CIN cervical intraepithelial neoplasia; FIGO Fédération Internationale de Gynécologie Obstétrique (International Federation of Gynecology and Obstetrics.*

**Table 5:** **Age-specific comparison of standard gamble utility scores converted using the ‘lifetime duration-early stage cervical cancer’ score**

Health state	Age groups				Wilcoxan rank sum test P value
	20-49 years		50-69 years		
	Mean utility score	Median utility score	Mean utility score	Median utility score	
	(SE)	(IQR)	(SE)	(IQR)	
Cyto normal	0.9996	1.0	0.9994	0.9999	0.9255
	(0.0002)	(0.9997 -1.0)	(0.0003)	(0.9998 -1.0)	
HPV vaccination	0.9998	1.0	0.9960	1.0	0.8408
	(<0.0000)	(0.9998 - 1.0)	(0.0034)	(0.9998 - 1.0)	
LG cyto	0.9996	1.0	0.9965	1.0	0.8174
	(0.0002)	(0.9997 -1.0)	(0.0032)	(1.0 -1.0)	
LG cyto with colp normal	0.9985	0.9999	0.9956	0.9999	0.8933
	(0.0086)	(0.9996 - 1.0)	(0.0032)	(0.9997 - 1.0)	
HPV positive with cyto normal	0.9986	0.9999	0.9956	0.9999	0.786
	(0.0009)	(0.9997 - 1.0)	(0.0032)	(0.9997 - 1.0)	
HPV positive with colp normal	0.9987	0.9999	0.9994	1.0	0.2892
	(0.0009)	(0.9997 - 1.0)	(0.0003)	(0.9998 - 1.0)	
Treated genital warts	0.9983	0.9998	0.9957	0.9999	0.8211
	(0.0010)	(0.9994 - 1.0)	( 0.0034)	(0.9997 - 1.0)	
HG cyto with CIN 1	0.9989	0.9999	0.9922	0.9998	0.8517
	(0.0007)	(0.9996 - 0.9999)	(0.0045)	( 0.9997 - 1.0)	
HG cyt with CIN 2 or 3	0.9983	0.9997	0.9954	0.9999	0.8733
	(0.0010)	(0.9994 - 0.9998)	(0.0033)	(0.9992 - 1.0)	
Early stage cervical cancer	0.9732	0.9950	0.9697	0.9850	0.4817
	(0.0085)	(0.9550 - 0.9950)	(0.0066)	(0.9450 - 0.9950)	

*HSPS health state preference score; Cyto cytology; HPV human papillomavirus; LG low grade disease; HG high grade disease; Colp colposcopy; CIN cervical intraepithelial neoplasia; FIGO Fédération Internationale de Gynécologie Obstétrique (International Federation of Gynecology and Obstetrics).*

## Discussion

This study is the first of its kind to assess cervical cancer prevention utility scores in a random sample of women from the general population (non-clinic based population) targeted for cervical screening. We assessed 10 hypothetical health states that include experiences for important future cervical screening strategies in an era of HPV vaccination that will potentially involve primary HPV testing. Although the sample size of the study was relatively small, we have generated a preliminary set of health state utilities that can be used to inform detailed cost-effectiveness evaluation of primary HPV testing, HPV testing with genotyping, co-testing, and cytology triage after HPV positive testing, in the context of the effect of HPV vaccination.

The utility scores for health states obtained in the current study that described the experience of having a normal cytology result and low grade cervical disease (with/without colposcopy) appeared to be similar to other studies which measured similar health states [7, 8, 15, 22, 23, 26]. For the health state of ‘a normal pap test result’, Kupperman et al. estimated a mean and median score of 0.989 and 1.0, respectively for women who were treated for a cervical abnormality [26], using a time trade-off instrument. This was broadly comparable to the range utility scores obtained in this study (0.9967 to 1.0). For low grade cytology (without colposcopy) the range of utility scores was between 0.9735 to 1.0, which were similar to a previous ‘two-stage standard gamble’ assessment conducted among women recruited through pharmacy and general practitioner clinics in Sydney, Australia (mean: 0.9972 and median: 0.9963) [15]. However, the values obtained in the current study were higher than from a study in family planning clinics in California (mean: 0.96) and a commonly used set of utilities from a study at Duke University hospital clinic (mean: 0.91 – it was assumed the reported score was a mean as opposed to median) [7, 8, 22]. It should be noted however, that the study in California involved hypothetical scenarios that involved repeated visits and additional smears and colposcopy, which may have influenced the values obtained, but it is not certain how scenarios were described since detailed vignette descriptions were not given.

For the cervical cancer health state measured in the current study, the range of utility scores obtained was between 0.8178 to 0.9900. These were similar to scores obtained from another study of women who underwent prior treatment for any abnormal cervical disease (mean 0.816 and median 0.972) [26]. By contrast, the range of health state preference scores for the health state ‘high grade cytology with a confirmed CIN 2/3’ obtained in this study (0.9704 to 0.9999) were significantly higher than those obtained from women who had a previous history of treatment for any cervical abnormality (mean: 0.835 and median: 0.984) and from women recruited for the Myers et al. study at Duke University hospital clinic (mean score for high grade squamous intraepithelial lesion: 0.91; mean score for CIN 2/3: 0.87 - it was assumed mean scores were reported) [7, 8, 26].

In the assessment of cervical cancer prevention health states, the use of the ‘two-stage standard gamble’ involves a shift in endpoints, that is, from the worst anchor state of ‘death’ to ‘early stage cervical cancer’. Therefore, in using our approach, a key assumption is that participants are able to shift the interpretation of the scale end points from the ‘early stage cervical cancer’ (low end) to ‘perfect health’ (high end) scale, to the ‘death’ (low end) to ‘perfect health’ (high end) scale [27]. If participants do not shift their endpoints in an evaluation, that is, they maintain the same risk attitude for assessments of non-cervical cancer health states towards the assessment of cervical cancer, the transformed health state preference score for non-cervical cancer health states will appear artificially high. Stalmeier has shown that when the worst anchor point has shifted, that is from ‘death’ to a worst temporary health state, the mathematically transformed health state preference score (as described and used in this study) produced higher outcomes compared with the conventional single stage assessment [27]. Given this observed phenomena in previous studies, it is possible that a potential lack of end-point adjustment by participants in the current study could explain the higher utility scores for some of the assessed health states, especially for the management of high grade disease.

In our study we observed that the two-stage standard gamble produced utility scores for health states that were generally lower when reported as means than when reported as medians. For example, the early stage cervical cancer utility score was 0.8178 when characterised using a mean (using ‘12 month’ time-in-state duration), whereas a median value indicated an average score of 0.9450. The reason for this is likely to reflect the small sample size and the wide distribution of scores. In this context, values that tend towards the lower end of a distribution are likely to pull the mean estimate down, whereas the median is not so affected by ‘outlier’ values.

The mean score can diverge from the median if there are a group of participants in the study sample who value an experience lower than other groups (due, for example to particular levels of underlying anxiety). For cost-effectiveness analysis, mean scores have been advocated by some health economists as they indicate the group strength of preference for a health state [21]. By contrast, the median value treats each health state valuation equally in a voting context and is less likely to be influenced by extreme values or outliers [21]. The decision to use either the mean or median is a matter of debate and thus different primary studies have used different methods [21]. Therefore, for interventions that are being evaluated for cost-effectiveness using decision analytic modelling, an array of health state preference scores needs to be assessed in order to determine whether the cost per QALY is affected by the choice of health state preference scores.

We also attempted to address the issue of whether different ‘time in state’ values for an anchor state used in a two-stage standard gamble has the potential to affect utility scores for temporary health states. Our results suggest that, according to the ICC values, there is the potential for ‘time in state’ to distort utility scores for temporary health states. On the other hand, this finding may be due to the small sample size obtained for our study. In the calculation of *MS*_*wthin grp*_ values, differences between individual utility scores and their mean can be large enough so as to provide a value that is similar to the respective *MS*_*btw grp*_ value; where the latter is based on the difference between two sets of utility scores (the mean of each ‘time in state’ specific health state utility score) and their (grand/overall) mean. Thus in the context of a small study, only a handful of larger differences between individual utility scores and their mean is required for an observation such as ours. However, a visual inspection of mean values for each set of temporary health states suggests that the differences in the utility scores are minor. Nonetheless, our findings do not preclude the need for such an issue to be investigated in the future. If ‘time in state’ values do have the potential to distort utility scores for temporary health states, this needs to be addressed appropriately in techniques that use ‘time in state’ (time-trade off) to derive utilities.

The limited number of women recruited into the study has the potential to affect the generalizability of our results. We sought to recruit women eligible for cervical screening in Australia (aged 18-20 to 69 years). This was done through recruiting a random sample of women living in metropolitan Sydney. However, given only 15.6% of women contacted agreed to be interviewed, there is the possibility that those who did not participate may have demographic, cultural, medical and/or lifestyle characteristics that are distinct from the sample obtained in this study. Selection bias refers to systematic error in the way women are recruited into the study. However, the NSW Department of Health Population Survey, from which our study participants were recruited, utilizes robust random sampling techniques to ensure the results of the survey accurately reflects the population of New South Wales. Thus, if the results of our survey are to be applied in cost-effectiveness analyses, they should be used in conjunction with other sources of health state preference scores so as to ensure a robust sample of women are represented from the population of interest.

Stated preferences for health states for cervical cancer prevention may be affected by the participant’s age at which the assessment is done. Such differences may occur, for example, for cervical screening with cytology, where a younger women, who may not have had a pap smear, could be more fearful of the procedure compared to an older women who has undergone the procedure on a regular basis. Consequently, the utility score attached to a health state may be lower amongst those who younger in the study population, relative to those who are older. As part of our analysis we explored whether this particular phenomena may occur amongst women aged 20-49 years relative to women aged 50-69 years. Our results did not find a statistically significant difference in the values attached to health states for cervical cancer between the two age groups. However, given our study was not powered to detect differences between these groups, the conclusion regarding this finding remains uncertain and requires further testing.

Differences between the rank of measured health state preference scores and those of ranking health states alone can potentially be explained by the instruments used; standard gamble versus ranking. The standard gamble utilises the notions of choice and uncertainty to evaluate health state preference values; whereas ranking is based on certainty of outcome in the absence of choice. Consequently, ranking does not reflect how decisions are made in the real world and are not considered reasonable proxies for estimating cardinal utility of health states. In our study, the ranking process required participants to focus on aspects of process, such as going through a colposcopy procedure. This state is ranked relative to other states. However, for the standard gamble participants are given a choice and are faced with the possibility of being diagnosed with cervical cancer if they do not engage in definitive preventive activities. Thus colposcopy may have been seen as an acceptable process to confirm the absence of disease, which could have been perceived as worthwhile, when compared to the probability of being diagnosed with cervical cancer. Therefore, the methods used to compare health state scenarios can influence the values assigned and the relative ranking of health states.

The utility set generated from the current study has already been employed in a supplementary analysis of QALYs performed as part of an evaluation of the cost-effectiveness of primary HPV testing in England [28] and Australia [29], and the utility set is also currently being used for similar supplementary analyses in government-commissioned assessments of primary HPV screening in Australia and New Zealand. Investigation of utilities related to primary HPV screening is a major research need in cervical cancer prevention policy evaluations since the choice of utility values has a considerable effect on the outcomes of such cost-effectiveness evaluations [29]. We have found, for example, that attaching a disutility to a screening test with a normal result (which can be broadly interpreted as ‘the experience of being screened’) has a profound impact on the relative cost-effectiveness of cervical screening strategies with different screening frequencies, as is the case for cytology vs. primary HPV strategies. Furthermore, because the values identified in the current study are associated with far less disutility (higher utility values) for diagnosis and management of screen-detected high grade abnormalities compared to the most commonly utilised set [7, 8], our findings potentially have important implications for the relative evaluation of different cervical screening technologies because different technologies are associated with different rates of high grade abnormalities in the population (due both to varying test characteristics and to the differing frequency of screening). Our findings for the disutilities associated with high grade abnormalities also have the potential to impact the evaluation of the relative benefits of vaccination and screening and thus the evaluation of the cost-effectiveness of vaccination itself (since vaccination in a cohort reduces the number of screen-positive events and their downstream sequelae experienced by that cohort).

## Conclusion

Our survey suggests health states relating to HPV testing are ranked lower than low grade cytology disease abnormalities. However, this did not translate into large differences in utility scores. Although in this initial assessment our sample size was limited, these results provide a preliminary set of population-based values (non-clinic) that may be used with other utility scores to explore the economic implications of introducing HPV testing as a primary screening tool in the context of HPV vaccination.

## Electronic supplementary material

Additional file 1: **Health state vignettes.** (DOCX 3 MB)
