# Supplementary material for: A survey of population-based utility scores for cervical cancer prevention
Source: BMC Res Notes. 2014 Dec 11;7:899. doi: 10.1186/1756-0500-7-899 (PMC4307910; doi:10.1186/1756-0500-7-899)
Supplement: Supplementary file 1 — Additional file 1: Health state vignettes. (DOCX 3 MB) [file 13104_2014_3471_MOESM1_ESM.docx]

**Supplementary material: Health state vignettes**
